# Correlation between Fine-Needle Aspiration Cytology and Histology for Palpable Breast Masses in a Nigerian Tertiary Health Institution

**DOI:** 10.1155/2015/742573

**Published:** 2015-10-08

**Authors:** Adetola Olubunmi Daramola, Mosebolatan Olatokunboh Odubanjo, Fred John Obiajulu, Nzechukwu Zimudo Ikeri, Adekunbiola Aina Fehintola Banjo

**Affiliations:** ^1^Department of Anatomic and Molecular Pathology, Lagos University Teaching Hospital and College of Medicine, University of Lagos, PMB 12003, Idi-Araba, Lagos, Nigeria; ^2^Department of Anatomic and Molecular Pathology, Lagos University Teaching Hospital, PMB 12003, Idi-Araba, Lagos, Nigeria

## Abstract

*Background*. Management of breast lumps can be challenging in resource poor settings. Fine-needle aspiration cytology (FNAC) especially when used with cell block can help improve affordability for the patients. *Objective*. To determine the diagnostic accuracy of FNAC of palpable breast lesions within a 5-year period. *Methods*. The findings obtained from FNAC of palpable breast lumps seen at the FNAC clinic of our department from January 2007 to December 2011 were retrieved and correlated with findings on histology of excisional biopsies. *Results*. A total of 1790 patients had FNAC of breast lumps during the 5-year period; 436 of them subsequently had biopsies. Our results compare favourably with the measures of test performance of the UK NHS Breast Screening Programme shown in brackets: absolute sensitivity 95.4% (>70%), complete sensitivity 99.2% (>90%), full specificity 88.9% (>65%), positive predictive value 99.6% (>99%), false-negative rate 0.8% (<4%), false-positive rate 0.4% (<0.5%), inadequate rate 3.2% (<15%), and suspicious rate 10.2% (<15%). *Conclusion*. Breast FNACs compare very well with histology of excisional biopsies and in experienced hands are extremely useful in the management of breast lumps. Further studies assessing the diagnostic accuracy of FNAC and cell blocks in our setting are recommended.

## 1. Introduction

Breast cancer is the commonest malignancy in women worldwide and the preoperative evaluation of breast lumps is an essential part of the management of breast lesions [1, 2]. The use of core needle biopsy in the management of palpable breast lumps in recent times has been increasing [3, 4]. This is because core needle biopsies are less invasive than open biopsy, and vacuum assisted biopsy devices have been developed to produce larger specimen for analysis [4]. Core needle biopsy is however not without disadvantages. These include a high cost (when compared with FNAC), long tissue processing time, patient discomfort such as pain and haematoma, and the risk of seeding of the tumor along the needle track [2, 5]. The triple test which comprises clinical, radiological, and pathological assessment however remains an excellent tool in the assessment of palpable breast lumps. Its diagnostic accuracy exceeds 99% when all three modalities are concordant [2, 6, 7].

Most countries have now adopted this triple assessment approach (clinical, radiological, and pathological) to breast diagnosis, with FNAC as the first-line pathological investigation in both screening and symptomatic populations, with the exception of cases where microcalcifications are present [8]. However, some variation in practice exists. When all three assessments are concordant, final treatment of malignant lesions (mastectomy, chemotherapy, and/or radiotherapy) may proceed on the basis of FNAC, without a tissue biopsy; some others insist on a core needle biopsy for all index lesions [8]. In most Nigerian hospitals, tissue biopsies are requested before mastectomy even when the lesion is malignant on FNAC, thus increasing the demand on scarce monetary resources. In a resource-limited setting such as ours, the case could be made for proceeding to mastectomy without requesting tissue biopsies, particularly if FNAC is combined with cell block preparations, to further increase its diagnostic accuracy and versatility. Smears and cell blocks can be tested for ER and PR using immunocytochemistry while HER2 testing can only be done by FISH as HER2 immunocytochemistry is unreliable even in the best centres.

FNACs, especially in the hands of experienced cytopathologists, have high diagnostic accuracy, as high as 98.9% in some series [9, 10]. Diagnostic accuracy of FNAC is further increased with cell block preparations approaching 100% in a particular study [11], making FNAC the most reliable element of the triple test in cases where the three modalities are nonconcordant [6].

In addition to its high diagnostic accuracy, FNAC offers advantages such as minimal invasiveness, minimal discomfort, cost-effectiveness, and rapidity of results when compared with core needle biopsy [5, 12, 13]. FNAC is therefore an extremely vital tool in the evaluation of palpable breast lumps in resource-limited settings.

The objective of this study is to determine the diagnostic accuracy of FNAC in the evaluation of palpable breast masses in LUTH.

## 2. Materials and Methods

Records of FNAC results of palpable breast lumps seen at the FNAC clinic of the Department of Anatomic and Molecular Pathology of the Lagos University Teaching Hospital (LUTH) from 2007 to 2011 were retrieved from the database. FNAC was performed for all the cases using a 23 G needle attached to 20 mL disposable plastic syringes smeared on standard microscope glass slides, fixed with alcohol, and stained with Haematoxylin and Eosin (H&E), modified Giemsa, and Papanicolaou (Pap) stains. These cases were reported using a 5-tier system: C1 for inadequate; C2 for benign; C3 for suspicious, probably benign; C4 for suspicious, probably malignant; and C5 for malignant breast lesions.

**Figure 1:**
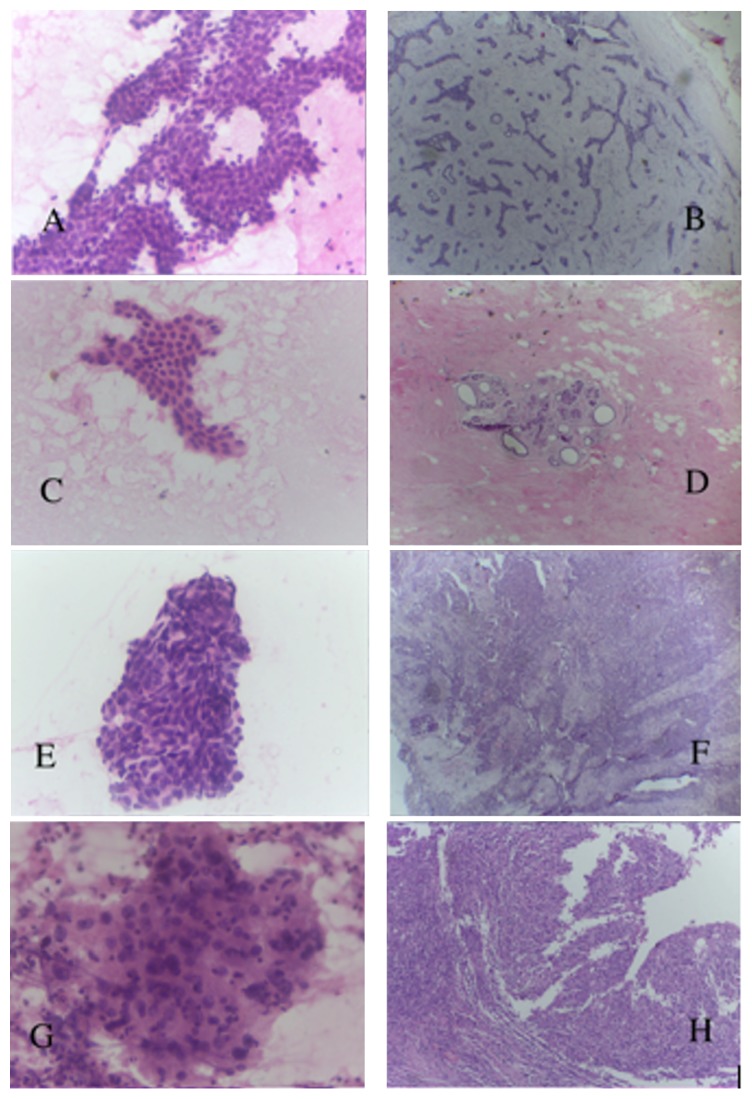
Photomicrograph showing smear preparations diagnosed as C2 (A), C3 (C), C4 (E), and C5 (G) and their corresponding diagnoses: fibroadenoma (B), fibrocystic changes (D), and invasive ductal carcinoma of no special type ((F) and (H)).

Records of subsequent excisional biopsies or mastectomies were also retrieved from the pathology database and compared with the cytology results for correlation (Figure 1). Quality assurance statistical parameters were calculated according to the NHS Breast Screening Programme (NHSBSP) guidelines [2].

Cases with missing data were excluded from the study.

## 3. Results

**Figure 2:**
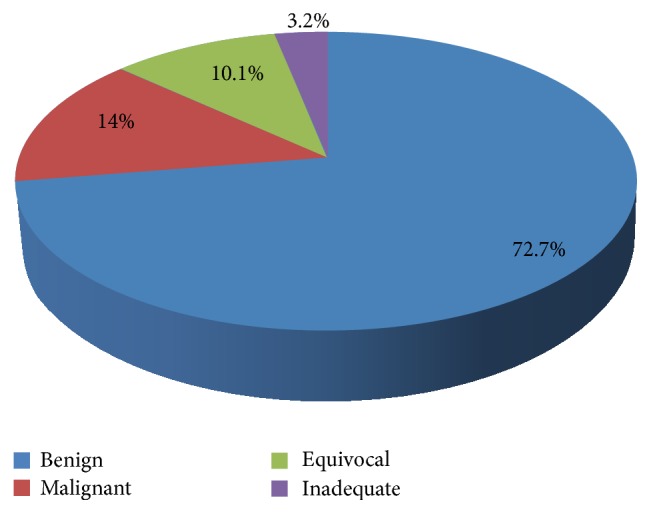
Spectrum of cytologic diagnosis.

**Figure 3:**
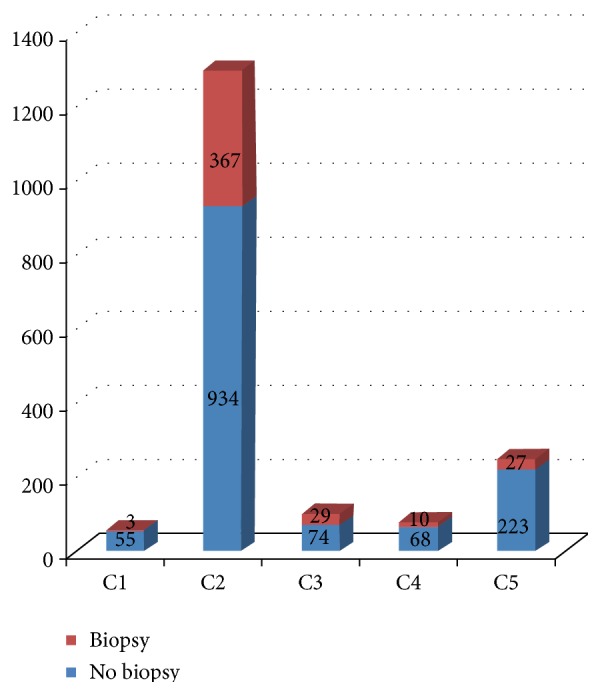
Cytology cases with follow-up biopsies.

FNAC was requested for a total of 1790 breast lumps during the study period. Of these, 1301 were benign, 250 were malignant, 181 were equivocal, and 58 were inadequate (Figure 2). Four hundred and thirty-six (436) of the 1790 cases subsequently had biopsies, a biopsy rate of 24.4% (Figure 3).

The correlation of cytological and histological diagnoses is shown in Table 1. Over 90% of cases from FNAC (96.3%) were confirmed to be malignant by histology, and 99.5% were confirmed to be benign. 82.8% suspicious but possibly benign on FNAC were found to be benign on histology, and 60% of cases suspected to be malignant were found to be malignant.

Quality assurance statistics were calculated according to the UK NHSBSP guidelines and the values are shown in Table 2. The absolute sensitivity, the number of carcinomas diagnosed as such on cytology expressed as a percentage of the total number of carcinomas sampled, was 95.4%. The complete sensitivity which is the number of carcinomas that were not definitely negative or inadequate on FNAC expressed as a percentage of the total number of carcinomas was 99.2%. Full specificity, which is the number of correctly identified benign lesions expressed as a percentage of the total number of benign lesions, was 88.9%. The positive predictive value, defined as the number of correctly identified cancers expressed as a percentage of the total number of positive results, was 99.6%.

Correlations of specific diagnoses made on cytology and histology are shown in Table 3. Of 8 cases suspected to be inflammatory on FNAC, 4 were confirmed by histology to be inflammatory, 1 was fibroadenoma, another was fibrocystic changes, 1 was hidrocystoma, and 1 was invasive lobular carcinoma. One case suspected to be a lipoma on cytology was confirmed to be lipoma on histology. The other case was found to consist of normal breast tissue only.

## 4. Discussion

FNAC is a useful tool in the preoperative evaluation of breast lumps [2]. Accurate preoperative evaluation is important as it allows for rapid referral of malignant cases for treatment and discharge of benign cases from the clinic and their return to routine follow-up [2]. FNAC can also be used in following up these benign cases except when otherwise indicated. FNAC is accurate, cheap, and easy to perform and is less invasive than core needle biopsies [5].

Quality assurance statistics calculated for this study (Table 2) compare favourably with the UK NHSBSP thresholds of performance and show a high accuracy of FNAC in evaluating breast lumps. Studies from other Nigerian centres also corroborate this finding. In a study done at the Aminu Kano University Teaching Hospital (AKTH), absolute sensitivity was 81%, specificity was 99%, and positive predictive value was 97.7% [14]. Another done in Benin reported an absolute sensitivity of 84.6%, complete sensitivity of 97.4%, full specificity of 64%, and positive predictive value of 100% [15]. These studies show the adequacy of FNAC in the evaluation of palpable breast lumps in resource-limited settings.

Our study showed a low frequency of inadequate specimens in line with the NHSBSP thresholds of performance. This is because of the on-site evaluation of the fine-needle aspiration specimens by pathologists using a rapid staining technique (modified Giemsa). On-site evaluation of aspirates has been shown to be accurate and cost-effective [13]. It also reduces the likelihood of being recalled for a repeat FNAC and therefore reduces delay in obtaining results [2].

Missing the lesion on sampling during aspiration is known to be the most common cause of a false-negative cytological diagnosis [2]. Certain carcinomas including lobular carcinoma (responsible for one of the false-negative results in this study, Table 3) may produce such a result [2]. Indeed, FNAC of invasive lobular carcinoma is associated with notoriously high rates of false-negative and equivocal diagnoses [16]. This is because classic invasive lobular carcinoma is more likely to yield a paucicellular smear with subtle atypia and rare single intact epithelial cells [16].

The false-positive case in this study was fibroadenoma with foci of proliferative changes. Fibroadenomas are the commonest cause of false-positive diagnosis in FNAC. This is because of the frequent presence of occasional isolated intact cells with dissociation, epithelial nuclear atypia, and high cellularity [17]. Apocrine metaplasia, multinucleation, and paucicellularity in hyalinized fibroadenomas are additional pitfalls [17].

In this study, 181 out of 1790 FNAC cases were equivocal, giving a suspicious rate of 10.1%. Though this value is within the preferred thresholds of performance, this figure can be reduced further by the use of cell block preparations which were not done in these cases. Cell blocks prepared from residual tissue fluid have been shown to assist in further establishing a more definitive cytopathologic diagnosis [18]. In one study, 3 cases suspicious for malignancy on FNAC were confirmed to be invasive ductal carcinoma with cell block [19]. Other limitations of breast FNAC that can readily be overcome by cell blocks are the difficulty in demonstrating invasion and classifying proliferative lesions [20]. ER and PR receptor testing can be reliably performed on both smears and cell blocks obtained from FNA using immunocytochemistry [8]. The results are expressed as the percentage of tumour cells showing nuclear staining while HER2 can only be reliably tested using fluorescent in situ hybridization (FISH).

## 5. Conclusion

Breast FNACs compare very well with histology for excisional biopsies, and, in experienced hands, they are extremely useful in the evaluation of breast lumps. Further studies assessing the diagnostic accuracy of FNAC and cell block preparations in our setting are in progress.

## Figures and Tables

**Table 1:** Correlation of cytological and histological diagnosis.

Cytology		Histology	
Diagnosis	Number with follow-up biopsies	Benign	Malignant
C1	3	3	0
C2	367	365	2
C3	29	25	4
C4	10	4	6
C5	27	1	26
Total	436	398	38

**Table 2:** Comparison of results with UK NHSBSP thresholds of performance.

Parameter	Minimum (%)	Preferred (%)	This LUTH study
Absolute sensitivity	>60	>70	95.4%
Complete sensitivity	>80	>90	99.2%
Full specificity	>55	>65	88.9%
Positive predictive value	>98	>99	99.6%
False-negative rate	<6	<4	0.8%
False-positive rate	<1	<0.5	0.4%
Inadequate rate	<25	<15	3.2%
Inadequate rate from cancers	<10	<5	0%
Suspicious rate	<20	<15	10.1%

**Table 3:** Correlation of specific cytologic and histologic diagnoses.

Cytology		Histology						
Diagnosis	Total number	Fibroadenoma	Inflammatory	Fibrocystic change	Normal breast	Lipoma	Hidrocystomas	Invasive lobular carcinoma
Fibroadenoma	6	6	—	—	—	—	—	—
Inflammatory	8	1	4	1	—	—	1	1
Fibrocystic change	2	—	—	2	—	—	—	—
Lipoma	2	—	—	—	1	1	—	—
Cystic breast lesions	1	1	—	—	—	—	—	—
